# Acute and Chronic Leukemia in Sub‐Saharan Africa: A Systematic Review

**DOI:** 10.1002/cnr2.70635

**Published:** 2026-07-31

**Authors:** Allwell A. Ayirebi, Stephen Twumasi, Linda K. Olaga, Benedict Sackey, Daniel N. M. Antonio, Richard O. Ansah, Eugene A. Ansah, John A. Sah, Enoch O. Anto, Yvonne Anang‐Quartey, Antoinette Koomson, Angela Opoku, Godfred A. Donkor, Paulina Duah, Ama G. Boadu, Samuel Essien‐Baidoo, Wina I. O. Boadu, Lilian A. Boateng

**Affiliations:** ^1^ Allwell Cancer Research Institute Kumasi Ghana; ^2^ Hematology Unit, Laboratory Division Kuntanase Government Hospital Bosomtwe Ghana; ^3^ Department of Medical Laboratory Technology Medicare College of Applied Sciences Kumasi Ghana; ^4^ Department of Medical Diagnostics Kwame Nkrumah University of Science and Technology Kumasi Ghana; ^5^ Department of Medical Laboratory Technology Garden City University Kumasi Ghana; ^6^ Department of Hematology Korle Bu Teaching Hospital Accra Ghana; ^7^ Hematology Unit, Central Laboratory Kumasi South Hospital Kumasi Ghana; ^8^ Genomics and Infectious Diseases Laboratory, Faculty of Allied Health Sciences, College of Health Sciences Kwame Nkrumah University of Science and Technology Kumasi Ghana; ^9^ Department of Molecular Medicine, School of Medicine and Dentistry Kwame Nkrumah University of Science and Technology Kumasi Ghana; ^10^ School of Medical and Health Sciences Edith Cowan University, Joondalup Drive Perth Australia; ^11^ Centre for Precision Health, ECU Strategic Research Centre Edith Cowan University Perth Australia; ^12^ Department of Physician Assistant Studies, School of Medicine University of Cape Coast Cape Coast Ghana; ^13^ Laboratory Unit Komfo Anokye Teaching Hospital Kumasi Ghana; ^14^ College of Health Sciences University of Ghana Medical School Accra Ghana; ^15^ Department of Rheumatology Korle Bu Teaching Hospital Accra Ghana; ^16^ School of Allied Sciences University of Health and Allied Sciences Ho Ghana

**Keywords:** acute leukemia, ALL, AML, chronic leukemia, CLL, CML, leukemia, sub‐Saharan Africa, systematic review

## Abstract

**Background:**

The four primary subgroups of leukemia include chronic myeloid leukemia (CML), acute myeloid leukemia (AML), acute lymphoblastic leukemia (ALL), and chronic lymphocytic leukemia (CLL). Very little is known about the regional variation of leukemia and subtypes in sub‐Saharan Africa (SSA).

**Aims:**

This systematic review focused on the prevalent cases reported in sub‐Saharan Africa, throwing more emphasis on treatment outcomes, follow up time and survival rates.

**Methods:**

A systematic review of studies on acute and chronic leukemia in sub‐Saharan Africa (SSA) was conducted using articles retrieved from PubMed, Scopus, Google Scholar, Embase and African‐specific databases such as African Journals Online (AJOL), covering publications from 1975 to July 15, 2025.

**Results:**

A total of 94 studies were subjected to initial screening from study titles. Thirty‐two (32) articles that met the inclusion criteria were selected for inclusion in this systematic review. Out of 2537 leukemia cases reported in the 32 studies, 1644 (64.80%) were acute and 893 (35.20%) were chronic. AML accounted for 947 (37.33%), 450 (17.74%) were ALL, 720 (28.38%) were CML, 173 (6.82%) were CLL, 2 (0.08%) were acute undifferentiated leukemia, 1 (0.04%) was mixed phenotype acute leukemia and 244 (9.62%) unclassified leukemias. Survival rates ranged from 2 weeks to 5 years. Fifty‐nine patients were lost to follow up and follow up time ranged from 11 months to 7 years. 273 (10.76%) leukemia related deaths were reported.

**Conclusion:**

This review found that acute leukemias were more prevalent than chronic leukemias, with AML emerging as the most frequently reported subtype. Survival durations ranged from a few weeks to five years; however, overall outcomes remained poor, characterized by high mortality rates and substantial loss to follow‐up. These findings underscore the urgent need for improved diagnostic capacity, standardized treatment protocols, and strengthened patient monitoring and follow‐up systems to enhance leukemia care and outcomes.

## 1 Introduction

Leukemia is a class of diverse cancers of hematopoietic stem cells (HSCs). It is typified by an abnormal buildup of immature or matured leucocytes that proliferate unchecked in the bone marrow, interfering with the synthesis of healthy blood cells. This malignancy includes chronic myeloid leukemia (CML), acute myeloid leukemia (AML), acute lymphoblastic leukemia (ALL), and chronic lymphocytic leukemia (CLL), which are the four primary subgroups of leukemia [1]. Acute leukemia, particularly AML and ALL, represents substantial challenges in the field of hematological cancers, and it affects a diverse age group [2]. AML is distinguished by the clonal proliferation of undifferentiated myeloid progenitors, known as blasts, inside the bone marrow compartment, and this proliferation is mostly due to the accumulation of various genomic and cytogenetic abnormalities. AML is a heterogeneous disease that necessitates personalized cytogenetic and molecular characterization [3]. The disease can be generally classified into favorable, moderate, or high‐risk groups based on the criteria given in the European Leukemia Net (ELN) 2022 recommendations for adult patients [4]. Genetic abnormalities that characterize favorable risk disease include chromosomal translocations t (8;21) (q22; q22.1) or inv. [16] (p13.1q22). Intermediate‐risk AML is diagnosed in the presence of any *FLT3‐ITD* mutation (diagnosed in the absence of adverse risk genetic lesions) or t (9;11) (p21.3; q23.3) or *KMT2A* rearrangement. High‐risk AML categorization can be diagnosed in the presence of several cytogenetic or molecular aberrancies, which notably include monosomy 5/del 5q or 7/deletion 7q, other monosomal or complex karyotypes (≥ 3 unrelated abnormalities), or mutations in *ASXL1*, *EZH2*, *SRSF2*, or *TP53* [3].

The clonal growth of myeloid progenitor cells in the bone marrow and peripheral blood is a hallmark of AML. The accumulation of various genomic and chromosomal abnormalities is the main cause of this proliferation, which leads to insufficient production of red blood cells and platelets due to poor erythropoiesis and megakaryopoiesis, organ infiltration, and bone marrow failure [5]. ALL is an aggressive cancer of B or T lymphoblasts characterized by the uncontrolled growth of aberrant, immature lymphocytes and their progeny. Like AML, the blastic lymphoid cells eventually replace functional bone marrow and lymphoid tissues, resulting in bone marrow failure. Notably, the sequestration of platelets and lymphocytes causes splenomegaly and hepatomegaly, respectively [6]. Furthermore, genetic investigations have found that somatic, polymorphic variations of *ARD5B*, *IKZF1*, and *CDKN2A* are linked to an elevated risk of ALL [7]. Other uncommon germline mutations in *PAX5*, *ETV6*, and especially p53 can greatly predispose to the development of leukemia [8]. Philadelphia chromosome (Ph)‐positive acute lymphoblastic leukemia (ALL) is an important molecular subtype associated with poor prognosis and increasing prevalence with age. The Philadelphia chromosome is identified in approximately 20%–30% of adults with ALL and about 5% of pediatric ALL cases, with the incidence increasing to nearly 50% among patients older than 50 years [9, 10]. In recent years, Philadelphia chromosome—like ALL (Ph‐like ALL) has emerged as a distinct high‐risk subtype of B‐cell precursor ALL characterized by a gene expression profile and frequent IKZF1 alterations like those observed in Ph‐positive ALL. The prevalence of Ph‐like ALL is estimated at approximately 12% in children, 21% in adolescents, and 20%–24% in adults older than 40 years, with the highest prevalence reported among young adults aged 21–39 years [11]. Additionally, Ph‐like ALL occurs more commonly in males, individuals with Down syndrome, and populations of Hispanic ancestry, and has been associated with inherited GATA3 genetic variants [11].

CML is a clonal hematopoietic stem cell disease with the Philadelphia chromosome [12], which is characterized by a reciprocal t (9;22) (q34; q11) chromosomal translocation, resulting in a 9q+ and a tiny 22q−. The BCR:ABL fusion gene, which produces a chimeric BCR‐ABL protein with unregulated tyrosine kinase activity, is essential for CML cell transformation [13]. In 2001, the FDA authorized Imatinib mesylate (Gleevec), a tyrosine kinase inhibitor (TKI), as a treatment for CML, which transformed the disease's therapeutic approach [14]. CLL is a malignancy of CD5+ B cells that causes the accumulation of small, mature neoplastic lymphocytes in the blood, marrow, and secondary lymphoid tissues, leading to lymphocytosis, leukemia cells in the marrow, lymphadenopathy, and splenomegaly. CLL is classified into two major subtypes, each with a distinct clinical presentation. CLL cells are classified based on whether they express an unmutated or mutated immunoglobulin heavy‐chain variable region gene (IGHV), indicating the normal B cell differentiation stage from which they originated [15]. CLL cells with unmutated IGHV are derived from B cells that did not differentiate in germinal centers. Germinal centers are lymph node sites where B cells undergo somatic hypermutation and selection during immunological response. Patients with CLL cells expressing an unmutated IGHV have a more aggressive illness than those with mutated or hypermutated IGHV.

Cancer disproportionately affects patients in low‐ and middle‐income countries, with 70% of newly diagnosed cancer cases happening in these regions, where cancer survival rates are 30% to 50% lower than those in high‐income countries [16]. This is due to population growth, limited screening and treatment, higher rates of infection‐related cancers, and weak healthcare systems. Socioeconomic barriers, lack of awareness, and poor infrastructure lead to late diagnoses and lower survival rates compared to high‐income countries, worsening global cancer inequalities. Although Africa accounts for only around 6% of the global cancer burden, it is not a rare disease on the continent. It imposes significant economic and social obligations on the inhabitants. To address this fast‐growing problem, Sub‐Saharan Africa (SSA) countries require a reasonable national cancer control strategy [17]. As a result, the African Cancer Registry Network (AFCRN) serves as the regional hub for the International Agency for Research on Cancer (IARC) in boosting our knowledge and resilience. Population‐based cancer registries' primary goals are to offer data on cancer incidence in that population and a framework for evaluating and managing the disease's effects on the community. Public health and epidemiology are the main objects of focus. In SSA, the five most common malignancies in males are prostate, liver, Kaposi sarcoma, esophageal, and colon cancer, while females have cervix uteri, breast, liver, colorectal, and ovarian cancers in decreasing order of age‐standardized occurrence [18]. Despite these statistics, hematological cancer incidence over a five [5] year period, according to the 2022 GLOBOCAN World Health Organization (WHO) report on SSA cancer statistics, demonstrated a prevalence of 4.2% of leukemic cases [19]. On average, these rates per year are low, which raises the question of whether there are actual low cases of disease development or diagnostic challenges, hypothetically. Therefore, this study aims to determine the reported proportion of acute and chronic leukemia, treatment regimens, and survival outcomes through a systematic review approach to advise policymakers and promote leukemia research in SSA.

## 2 Study Design and Methods

### 2.1 Study Design

This systematic review is reported according to the 2020 Preferred Reporting Items for Systematic Reviews and Meta‐Analyses (PRISMA) statement [20]. The protocol for the study has been registered in PROSPERO with the following ID number: CRD420251025330.

### 2.2 Search Strategy

A comprehensive search strategy was developed in consultation with the Preferred Reporting Items for Systematic Reviews and Meta‐Analyses (PRISMA) 2020 guidelines to maximize the identification of relevant studies. Electronic searches were conducted in PubMed/MEDLINE, Scopus, Embase, Google Scholar, and African Journals Online (AJOL) from database inception to January 15, 2025. The search strategy combined controlled vocabulary (e.g., Medical Subject Headings [MeSH], where applicable) and free‐text terms using Boolean operators (AND/OR). Search terms included general disease terms (“leukemia” and “leukaemia”), disease‐specific subtypes and abbreviations (“acute myeloid leukemia” [AML], “acute lymphoblastic leukemia” [ALL], “chronic myeloid leukemia” [CML], and “chronic lymphocytic leukemia” [CLL]), geographic terms related to sub‐Saharan Africa, including regional and country‐specific names where appropriate, and treatment‐ and outcome‐related terms such as chemotherapy, radiotherapy, stem cell transplantation, remission, survival, mortality, morbidity, and quality of life. Database‐specific syntax and field tags were adapted for each database to optimize retrieval. Complete reproducible search strategies, including search strings, filters, limits, and date restrictions for each database, are provided in Appendix. Additionally, reference lists of the identified studies were manually examined to determine additional eligible studies. After removal of duplicates, using EndNote, results were screened by their titles and non‐relevant articles were removed. Remaining articles were independently reviewed by their abstracts or full text by two authors (AAA and ST) and selected according to the pre‐specified inclusion criteria stated below. Discrepancies identified during the review process were resolved through discussion and consensus among the authors.

### 2.3 Inclusion Criteria

All studies published in peer‐reviewed journals, without restriction to language and study design, that reported data on adults and children with acute or chronic leukemia (including ALL, AML, CLL, CML) in SSA were considered for inclusion. Both observational studies (cohort, case–control, cross‐sectional) and clinical trials on chronic and acute leukemia in SSA were included. We ran the most recent search on July 15, 2025. The inclusion criteria for all studies were based on the PICO (Participant, Intervention, Comparison, Outcome) framework: P: Adults and children with acute or chronic leukemia in sub‐Saharan Africa; I: Various treatment regimens (chemotherapy, stem cell transplantation, radiotherapy, etc.); C: Different treatment options or regional healthcare systems, chronic versus acute leukemia outcomes; O: Survival rates, remission rates, morbidity, mortality, and quality of life.

### 2.4 Exclusion Criteria

We excluded studies on non‐Sub‐Saharan African populations, leukemia‐like conditions (e.g., myelodysplastic syndromes), and studies with insufficient data on key outcomes (e.g., incidence, survival, etc.).

### 2.5 Data Extraction

Two authors (AAA and ST) independently screened all titles and abstracts retrieved by the search against the selection criteria and obtained full texts when necessary. We extracted data using Microsoft Excel (Microsoft, Redmond, Washington, United States). Screening included study design, methods (country, setting, authors, study and publication years, study design), age, sex, number of participants, type, frequency and specificities of leukemia (acute vs. chronic, myeloid vs. lymphoid), pre‐existing conditions (HIV, malnutrition etc.), Treatment option (chemotherapy, bone marrow transplant, targeted therapy, etc.), treatment protocol (details of chemotherapy regimens, drugs used, frequency/dosage, or specific procedures (e.g., stem cell transplants)), duration of treatment (how long were participants treated or followed up?) and outcome assessment (survival rate/mortality rate, and follow‐up duration).

### 2.6 Quality Assessment

In order to assess the methodological quality and internal validity of the considered studies, a modified version of the Quality Assessment Tool for Observational Cohort and Cross‐Sectional Studies developed by the National Heart, Lung and Blood Institute, NHLBI [21] (Table 1) was applied independently by two authors (AAA and ST).

**TABLE 1:** Quality assessment of the 32 studies on leukemia.

Study (year)	Outcomes of the quality assessment criteria													
	1	2	3	4	5	6	7	8	9	10	11	12	13	14
Amsel (1975)	NR	+	+	±	NR	+	+	+	+	+	+	NA	NR	NA
Kusman (1976)	+	+	+	±	NR	+	+	+	+	+	+	NA	+	NA
Jacobs (1983)	+	+	+	±	NR	+	+	+	NR	NR	+	NA	−	NA
Jacobs (1985)	+	+	+	+	NR	+	+	+	±	±	+	NA	NR	NA
Jacobs (1990)	+	+	+	±	NR	+	+	+	+	+	+	NA	NR	NA
Aken'ova (1993)	+	+	+	±	NR	+	+	+	+	+	+	NA	NR	NA
Omoti (2010)	+	+	+	+	NR	NR	NR	NR	NR	NR	NR	NA	NR	NR
Chagaluka (2013)	+	+	+	±	NR	+	+	+	+	+	+	NA	NR	NA
Chukwu (2015)	+	+	−	±	NR	−	−	−	−	−	−	NA	NR	NA
Sissolak (2015)	+	+	+	+	NR	+	+	+	+	+	+	NA	−	NA
Faye (2016)	+	+	+	+	NR	+	+	+	+	+	+	NA	+	NA
Tapela (2016)	+	+	+	±	NR	+	+	+	+	+	+	NA	+	NA
Rubagumya (2017)	+	+	+	±	NR	+	+	+	+	+	+	NA	+	NA
Egesie (2018)	+	+	+	+	NR	NR	NR	NR	NR	NR	NR	NA	NA	NR
Fentie (2019)	+	+	+	±	NR	+	+	+	+	+	+	NA	+	NA
Aworanti (2020)	+	+	+	±	NR	±	±	±	±	±	±	NA	−	NA
Akaba (2020)	+	+	+	+	NR	NR	NR	NR	NR	NR	NR	NA	NA	NR
Owojuyigbe (2020)	+	+	+	=	NR	NR	NR	NR	NR	NR	NR	NA	NR	NR
Osei‐Owusu (2021)	+	+	+	±	NR	NR	+	NR	NR	NR	±	NA	−	NA
van Weelderen (2022)	+	+	+	±	NR	+	+	+	+	+	+	NA	+	NA
Shein (2021)	+	+	+	+	NR	+	+	+	+	+	+	NA	+	NA
Olbara (2021)	+	+	+	+	NR	NR	NR	NR	NR	NR	+	NA	NR	NA
Kloopers (2021)	+	+	+	+	NR	NR	+	NR	NR	NR	+	NA	+	NA
Musaigwa (2021)	+	+	+	±	NR	NR	+	NR	NR	NR	+	NA	+	NA
Kasonkanji (2022)	+	+	+	±	NR	+	+	+	+	NR	+	NA	−	NA
Hodkinson (2022)	+	+	+	±	NR	+	+	+	+	+	+	NA	+	NA
Bachi (2022)	+	+	+	+	NR	NR	NR	NR	NR	NR		NA	NA	NR
Jenkins (2023)	+	+	+	+	NR	±	+	−	+	−	+	NA	NR	NA
Naicker (2023)	+	+	+	+	NR	±	+	+	+	+	+	NA	+	NA
Vaughan (2024)	+	+	+	±	NR	+	+	+	+	NR	+	NA	NR	NA
Hodkinson (2024)	+	+	+	±	NR	NR	+	NR	NR	NR	+	NA	+	NA
Orido (2025)	+	+	+	+	NR	NR	NR	NR	NR	NR	NR	NA	NA	NR

*Note:* “+” indicates that all criteria mentioned are met; “±” indicates that the main or most important criteria is met, but does not meet all mentioned criteria; “−” indicates that the mentioned criteria are not met. Criteria: 1—Was the research question or objective in this paper clearly stated? 2—Was the study population clearly specified and defined? 3—Was the participation rate of eligible persons at least 50%? 4—Were all the subjects selected or recruited from the same or similar populations (including the same time period)? Were inclusion and exclusion criteria for being in the study prespecified and applied uniformly to all participants? 5—Was a sample size justification, power description, or variance and effect estimates provided? 6—For the analyses in this paper, were the exposure(s) of interest measured prior to the outcome(s) being measured? 7—Was the timeframe sufficient so that one could reasonably expect to see an association between exposure and outcome if it existed? 8—For exposures that can vary in amount or level, did the study examine different levels of the exposure as related to the outcome? 9—Were the exposure measures (independent variables) clearly defined, valid, reliable, and implemented consistently across all study participants? 10—Was the exposure(s) assessed more than once over time? 11—Were the outcome measures (dependent variables) clearly defined, valid, reliable, and implemented consistently across all study participants? 12—Were the outcome assessors blinded to the exposure status of participants? Not applicable for our study. 13—Was loss to follow‐up after baseline 20% or less? 14—Were key potential confounding variables measured and adjusted statistically for their impact on the relationship between exposure(s) and outcome(s)? Not applicable for our study.

Abbreviations: NA: not applicable; NR: not reported.

## 3 Results

### 3.1 Literature Search

**FIGURE 1:**
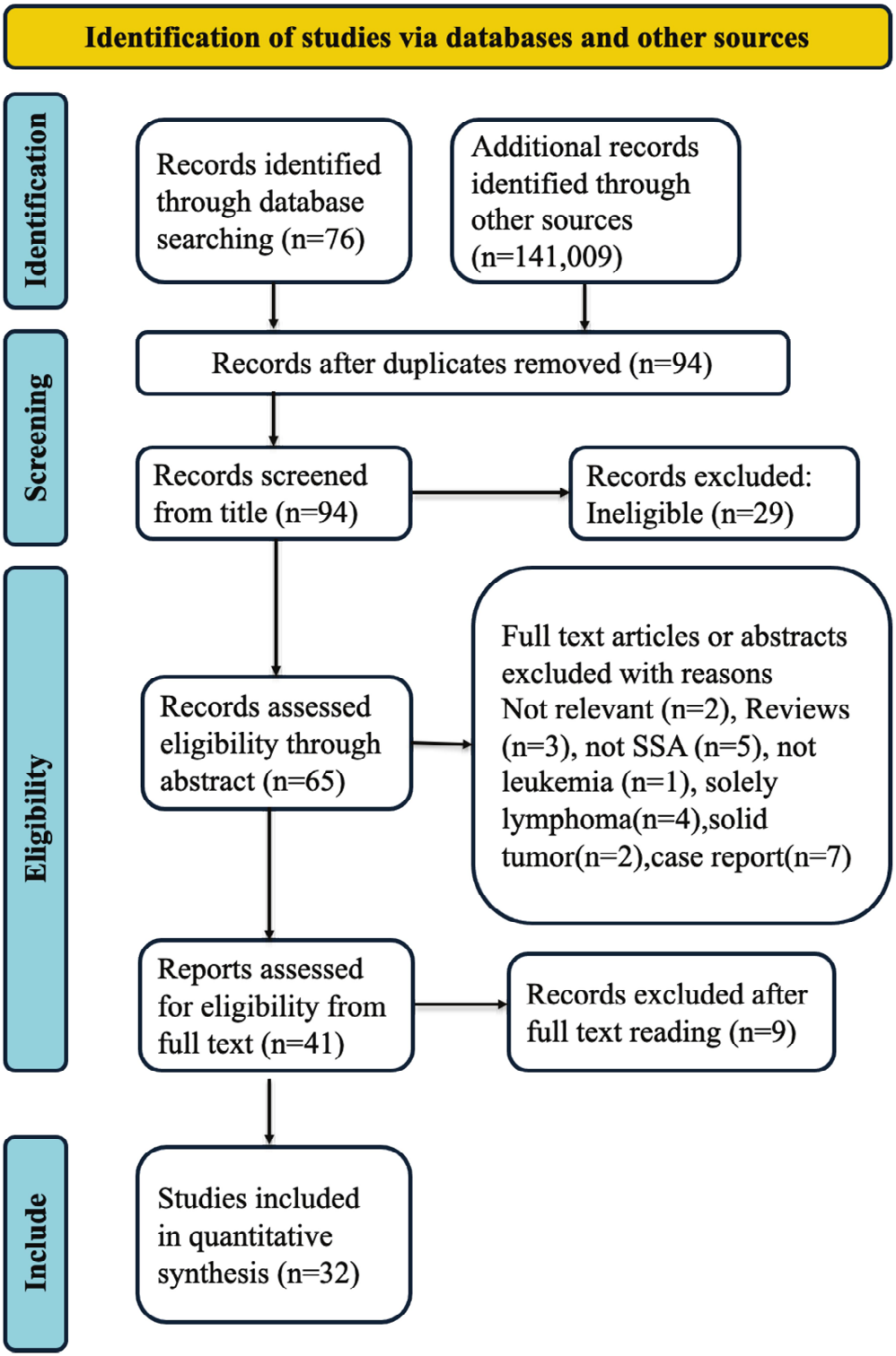
PRISMA flow diagram.

A total of 94 studies were subjected to initial screening from study titles, following which 65 studies were selected for a detailed screening process and 29 studies were excluded. From the selected 65 studies, an additional 24 studies were excluded due to other reasons, as shown in Figure 1. Nine of the remaining 41 studies were excluded from analyses after full‐text reading. Finally, 32 articles [22, 23, 24, 25, 26, 27, 28, 29, 30, 31, 32, 33, 34, 35, 36, 37, 38, 39, 40, 41, 42, 43, 44, 45, 46, 47, 48, 49, 50, 51, 52, 53] that met the inclusion criteria were selected for inclusion in the systematic review (Figure 1).

### 3.2 Study Quality Assessment

A total of 32 studies were assessed using 14 predefined methodological quality criteria as indicated in Table 1. The majority demonstrated clear research objectives (Criterion 1) and adequately defined their study populations (Criterion 2). For instance, studies by Faye et al. [30], Shein et al. [49], and Naicker et al. [42] were exemplary in explicitly stating their aims and specifying participant characteristics. Furthermore, most studies ensured consistent recruitment from similar populations and applied inclusion/exclusion criteria uniformly (Criterion 4), as seen in the works of Kusman et al. [40], Tapela et al. [51], and Vaughan et al. [53]. Exposure and outcome variables were generally well defined and implemented consistently (Criteria 9 and 11). Studies such as van Weelderen et al. [52] and Hodkinson et al. [32] met these standards, employing reliable measurement techniques and maintaining methodological consistency across participants. However, exposure assessment over time (Criterion 10) and evaluation of varying levels of exposure (Criterion 8) were less commonly addressed. Temporal alignment of exposure and outcome (Criterion 6) was met in most cases, albeit not always explicitly described. Studies such as Jenkins et al. [37] and Chagaluka et al. [26] outlined timelines that allowed for reasonable inference of association, though others like Chukwu et al. [27] and Aworanti et al. [25] lacked sufficient detail. Adjustment for confounding variables and blinding of outcome assessors (Criteria 12 and 14) were not applicable to the majority of studies included in this review due to their observational or descriptive nature. Nonetheless, more recent publications—such as those by Musaigwa et al. [41] and Hodkinson et al. [33]—demonstrated higher overall reporting standards, suggesting gradual methodological improvements over time. In summary, the included studies ranged from moderate to high methodological quality (Table 1).

### 3.3 Study Characteristics

The 32 articles were published between 1975 and July 15, 2025 and involved 2537 reported cases of leukemia. The geographical distribution included 9 of the 49 SSA countries: 13 studies were from South Africa, 8 from Nigeria, 3 from Kenya, 2 each from Malawi and Rwanda, and 1 each from Uganda, Senegal, Ghana, and Ethiopia. Study duration ranged from 1 year to 21.7 years. The median age of patients was 20.5 (IQR 0.5–66.9) years and a mean of 22.8 (SD 18.8) years (Table 2).

**TABLE 2:** General characteristics of the studies ordered by publication year.

First author	Year	Country	Study duration	Study design	Median age	Age range	Male	Female	Cases	Pre‐existing condition
Amsel [24]	1975	Uganda	Nov, 1971–Aug, 1973	P	5	0.5–15	14	3	23	NR
Kusman [40]	1978	South Africa	Oct, 1973–Sept, 1975	P	5.9/32	NR	17	17	34	NR
Jacobs [35]	1983	South Africa	NR	R	NR	NR	54	38	92	NR
Jacobs [34]	1985	South Africa	Jan, 1980–Jun, 1984	P	19	3.0–69.0	NR	NR	73	NR
Jacobs [36]	1990	South Africa	Jan, 1980–Jun, 1984	P	24	10.0–69.0	NR	NR	85	NR
Aken'ova [23]	1993	Nigeria	Jan, 1987–Dec, 1990	P	NR	NR	17	8	25	NR
Omoti [44]	2010	Nigeria	Jul, 2004–Jun, 2008	P	NR	20–70	32	15	47	NR
Chagaluka [26]	2013	Malawi	Dec, 2009–Aug, 2011	P	7.3	NR	11	9	20	HIV
Chukwu [27]	2015	Nigeria	Jan, 2011–Dec, 2013	P	NR	0–17	NR	NR	16	NR
Sissolak [50]	2015	South Africa	Jan, 2003–Feb, 2012	P	≥ 16	NR	34	24	58	HIV, diabetes, heart diseases, GIT carcinoma
Faye [30]	2016	Senegal	Jan, 2008–Feb, 2015	P	42	11.0–74	34	21	55	NR
Tapela [51]	2016	Rwanda	Jul, 2009–Jul, 2014	P	36.9	29–42	17	14	43	NR
Rubagumya [48]	2017	Rwanda	Jul, 2012–Dec, 2015	R	10	0.38–40.35	23	29	42	HIV positive (5%)
Egesie [29]	2018	Nigeria	Jan, 2005–Dec, 2015	R	NS	18–65	NS	NS	31	NS
Fentie [31]	2019	Ethiopia	Oct, 2016–Sept, 2017	P	36	14–74	87	60	147	Cytopenia
Aworanti [25]	2020	Nigeria	Jan, 2011–Dec, 2017	P	—	—	—	—	58	Sickle cell disease
Akaba [22]	2020	Nigeria	2010–2019	R	59	40–82	24	23	47	NR
Owujuyigbe [47]	2020	Nigeria	Nov, 2004–May, 2017	P	38	10–87	124	106	230	NR
Osei‐Owusu [46]	2021	Ghana	NR	P	9.88	7.02–12.74	14	12	26	NR
van Weelderen [52]	2022	Kenya	Jan, 2010–Dec, 2018	R	8.6	0–16	41	32	73	NR
Shein [49]	2021	South Africa	Jan, 1998–Aug, 2019	R	30	14–73	NR	NR	69	HIV; Coagulopathy Bleeding
Olbara [43]	2021	Kenya	Jan, 2010–Dec, 2016	R	7.8	4.8–11.2	73	63	136	NR
Kloopers [39]	2021	South Africa	Nov, 2018–Dec, 2019	P	38.5	18–85	NR	NR	40	NR
Musaigwa [41]	2021	South Africa	Jan, 2011–Dec, 2016	R	66.79	37–95	41	39	80	HIV
Kasonkanji [38]	2022	Malawi	Jun, 2013–Dec, 2019	P	19	15–39	13	6	19	CNS involvement tuberculosis
Hodkinson [32]	2022	South Africa	Apr, 2016–Oct, 2019 (42 M)	R	34	NR	12	10	22	HIV Positive (*n* = 10)
Dachi [28]	2022	Nigeria	Jan, 2018–Dec, 2020	R	22 ± 9.2	6 M–60 Y	22	7	29	NR
Jenkins [37]	2023	South Africa	Jan, 2005–Dec, 2018	R	50	17–84	28	28	218 [56]*	NR
Naicker [42]	2023	South Africa	Jan, 2015–Dec, 2019	R	31.8	15–65	15	12	27	Hypertension, HIV, pulmonary tuberculosis, diabetes mellitus, asthma, mitral valve prolapses and renal failure.
Vaughan [53]	2024	South Africa	2016–2019 (42 M)	R	37.5	17–58	143	134	461	HIV positive (*n* = 23)
Hodkinson [33]	2024	South Africa	Jan, 2019–Dec, 2022	R	427	6–62	NR	NR	194	NR
Orido [45]	2025	Kenya	NR	R	NR	NR	NR	NR	17	NR

Abbreviations: NR = not reported; NS =  not specified; *P* = prospective; *R* = Retrospective.

* Actual cases of which the study focused on are in parenthesis.

### 3.4 Types of Reported Leukemia

Out of 2537 leukemia cases reported in the 32 studies, 1644 (64.80%) were acute, and 893 (35.20%) were chronic. AML accounted for 947 (37.33%), 450 (17.74%) were ALL, 720 (28.38%) were CML, 173 (6.82%) were CLL, 2 (0.08%) were acute undifferentiated leukemia, 1 (0.04%) was mixed phenotype acute leukemia, and 244 (9.62%) were unclassified leukemias (Table 3). By country, the highest number of cases was reported from South Africa with 1453 cases, followed by Nigeria with 483 cases and Kenya with 226 cases. Moderate numbers of cases were reported from Ethiopia (147) and Rwanda [85], while relatively fewer cases were documented in Senegal [55], Malawi [39], Ghana [26], and Uganda [23].

**TABLE 3:** Clinical characteristics of study participants ordered by authors.

First author	Cases	CLL	ALL	CML	AML	Mixed phenotype acute leukemia	Acute undifferentiated leukemia	Morphological classification	Phenotypical classification	Subtype	Acute leukemia	Chronic leukemia
Amsel^√^	23	—	17	—	—	—	—	NR	NR	NR	23	—
Kusman	34	—	10	—	22	—	2	FAB M1, M2, M3, M4, M5, M6	NR	NR	34	—
Jacobs	92	—	—	92	—	—	—	NR	NR	NR	—	92
Jacobs**	73	15	58		—	—	—	NR	Common ALL (17), Null‐ALL (17), T‐ALL (18), B‐ALL (6)	NR	58	15
Jacob	85	—	85	—	—	—	—	FAB L1 & L2	NR	NR	85	—
Aken'ova	25	—	0	25	—	—	—	NR	NR	NR	—	25
Omoti	47	19	5	14	9	—	—	NR	NR	NR	14	33
Chagaluka	20	—	20	—	—	—	—	NR	NR	NR	20	
Chukwu*	16	—	10	1	5	—	—	NR	NA	NR	15	1
Sissolak	58	—	—	58	—	—	—	NR	NR	CP	—	58
Faye	55	—	—	55	—	—	—	NR	NR	CP: 85.5%;AP: 12.7%; BC: 1.8%	—	55
Tapela	43	—	—	43	—	—	—	NR	NR	NR	—	43
Rubagumya	42	—	42	—	—	—	—	NR	B‐cell (9); T‐cell (8); Unknown (25)	NR	42	—
Egesie	31	12	2	16	—	—	—	NR	NR	NR	3	28
Fentie	147	—	—	147	—	—	—	NR	NR	CP‐CML (136), AP‐CML (11)		147
Aworanti^†^	58	—	1	—	3	1	—	NR	NR	NR	58	—
Akaba	47	47	—	—	—	—	—	NR	B‐cell	NR	—	47
Owujuyigbe	230	—	—	230	—	—	—	NR	NR	NR	—	230
Osei‐Owusu	26	—	26	—	—	—	—	NR	NR	NR	26	—
van Weelderen	73	—	—	—	73	—	—	NR	NR	NR	73	—
Shein	69	—	—	—	69	—	—	Typical‐46; Microgranular‐9	NR	PMR‐RARA (21); Subtype 1 (13), 2 (3), 3 (5)	69	—
Olbara	136	—	136	—	—	—	—	NR	NR	NR	136	—
Kloopers	40	—	—	—	40	—	—	—	—	—	40	—
Musaigwa	80	80	—	—	—	—	—	—	—	—	—	80
Kasonkanji	19	—	19	—	—	—	—	NR	B‐ALL (8), T‐ALL (11)	NR	19	
Hodkinson	22	—	—	22	—	—	—	—	Myeloid BC‐CML (16), Lymphoid BC‐CML (4)	BC‐CML	—	22
Dachi	29	—	19	—	10	—	—	FAB (L1‐6. L2‐11, L3‐2) FAB (M1‐1. M2‐5, M3‐1, M4‐2, M5‐1)	—	—	29	—
Jenkins	218 (56)	—	—	—	218 (56)	—	—	—	—	CN‐AML (56)	218	—
Naicker	27	—	—	—	27	—	—	Typical‐11, Microgranular‐15	—	—	27	—
Vaughan^$^	461	—	—	—	277	—	—	—	—	—	461	—
Hodkinson	194	—	—	—	194	—	—	NR	NR	NR	194	—
Orido	17	—	—	17	—	—	—	NR	NR	NR	—	17

Abbreviations: ALL = acute lymphoblastic leukemia; AML = acute myeloblastic leukemia; AP = Accelerated phase; B‐ALL = B‐cell acute lymphoblastic leukemia; BC = blast crises; CLL = chronic lymphocytic leukemia; CML = chronic myelocytic leukemia; CP = chronic phase; FAB = French American British Classification; NR = not reported; T‐ALL = T‐cell acute lymphoblastic leukemia.

* reported on lag time and Diagnostic time.

^†^ Out of 58 acute leukemia patients, only 5 who had SCD were described and thus was included in the study. The acute leukemia population was 58.

^√^ Out of 23 acute leukemia patients, 17 were ALL patients and thus were included in the study. The acute leukemia population was 23.

** Excluded 15 CML cases from the study, but this has been included in the current review.

^$^ 461 acute leukemia cases, 277 AML were analyzed.

The distribution of cases showed that leukemia studies spanned from 1975 to 2025. Earlier years such as 1975, 1976, 1983, 1985, 1990, and 1993 recorded relatively few cases. However, there was a marked increase in reported cases from 2019 onwards, with the highest number recorded in 2024 (655 cases), followed by 2021 (424 cases), 2020 (335 cases), and 2023 (245 cases). This trend suggests increasing research activity and improved leukemia diagnosis and reporting in recent years. Regarding study design, retrospective studies accounted for most cases (1538), whereas prospective studies contributed 999 cases. (Table 4).

**TABLE 4:** Summary of leukemia cases grouped by country, year, study design, and leukemia subtype.

Group	Cases
*Country*	
South Africa	1453
Ethiopia	147
Ghana	26
Kenya	226
Malawi	39
Nigeria	483
Rwanda	85
Senegal	55
Uganda	23
*Year*	
1975	23
1976	34
1983	92
1985	73
1990	85
1993	25
2010	47
2013	20
2014	16
2015	58
2016	98
2017	42
2018	31
2019	147
2020	335
2021	424
2022	70
2023	245
2024	655
2025	17
*Study design*	
Prospective	999
Retrospective	1538
Leukemia sub‐type	
Acute leukemia	1644
AML	947
ALL	450
Acute undifferentiated	2
Mixed phenotype acute	1
Chronic leukemia	893
CML	720
CLL	173
Unclassified leukemia	17

### 3.5 Treatment Regimens

Different treatment regimens were reported depending on the type of leukemia (Table 5). Earlier studies, such as those by Amsel [24] and Kusman et al. [40], predominantly used classical chemotherapeutic agents including vincristine, prednisone, daunorubicin, cytarabine, and intrathecal methotrexate for induction and CNS prophylaxis in acute leukemia. Kusman et al. [40] provided one of the most comprehensive regimens, incorporating induction, consolidation, CNS prophylaxis, maintenance, and systematic reinduction phases. For acute lymphoblastic leukemia (ALL), treatment regimens evolved from multi‐agent chemotherapy protocols (e.g., vincristine, prednisone, L‐asparaginase, methotrexate) [34, 36] to resource‐adapted protocols such as the Hunger regimen [48] and the modified CALGB 10403 regimen [38]. These regimens typically included induction, consolidation, maintenance, and CNS prophylaxis components. Acute promyelocytic leukemia (APL) was treated using the PETHEMA LPA99 protocol [49] and ATRA‐based regimens in combination with anthracyclines [42]. Also, Jenkins et al. [37] documented the use of allogeneic stem cell transplantation for cytogenetically normal AML (CN‐AML). For chronic myeloid leukemia (CML), early management approaches included splenic radiotherapy and oral busulphan, as reported by Jacobs et al. [35] and Aken'ova et al. [23]. In more recent studies, tyrosine kinase inhibitors (TKIs) such as imatinib were utilized as first‐line therapy, whereas dasatinib and nilotinib were frequently utilized as second‐line therapies [30, 31, 47, 50, 51].

**TABLE 5:** Treatment regimens reported in selected studies.

First author	Treatment regimen
Amsel	ALL: Induction: IV vincristine 1.5–2 mg/m^2^ weekly for 4–6 weeks, oral prednisone 40–60 mg/m^2^ daily for 4–6 weeks. Allopurinol was given during the treatment period.
Kusman^a^	Allopurinol given before chemotherapy. AML: Induction (maximum of 6 courses): 15 mg/kg IV daunorubicin on day 1, 10 mg/kg IV cytosine arabinoside on day 1–5, 12 hourly. CNS prophylaxis: cranial irradiation with 2400 rad and intrathecal methotrexate 12 mg/m twice weekly for 5 days. Maintenance: IV 2 mg/m^2^ vincristine on day 1, oral 100 mg/m^2^ cyclophosphamide on days 1–5, IV 100 mg/m^2^ cytosine arabinoside on days 1–5 and oral 100 mg prednisolone twice daily on day 1–5 or 60 mg/m^2^ thioguanine daily. ALL and acute undifferentiated leukemia: Induction: IV 2 mg/m^2^ vincristine weekly for 3 weeks, oral 40 mg/m^2^ prednisone daily for 3 weeks CNS prophylaxis: cranial irradiation with 2400 rad was given and intrathecal methotrexate 12 mg/m^2^ on alternate days for 6 days. Maintenance: 6‐mercaptopurine 50 mg/m^2^/d by mouth, methotrexate 20 mg/m^2^ by mouth weekly, and cyclophosphamide 200 mg/m^2^ by mouth weekly. Systematic reinduction (every 10 weeks): vincristine 2 mg/m IV weekly for 3 weeks and prednisolone 40 mg/m by mouth for 2 weeks.
Jacobs	CML: Splenic radiotherapy and adjuvant busulphan (Myleran)
Jacobs	ALL: The adults were managed with conventional doses of vincristine, prednisone, I‐asparaginase and adriamycin. Those achieving complete remission underwent craniospinal prophylaxis and entered a 3‐year maintenance programme of oral 6‐mercaptopurine and methotrexate with monthly drug intensification. The children were stratified into low or high risk; the former were treated with a St Jude regimen [100] and the latter with a modified LSA‐L2 protocol [101].
Jacobs	ALL: Induction: Weekly intravenous infusions of vincristine (2 mg), Adriamycin (25 mg/m^2^), I‐asparaginase (10 000 units/m^2^), and daily oral prednisone (1 mg/kg). Consolidation was given immediately after remission had been achieved, using the identical drug regimen for an additional 4 weeks. CNS prophylaxis consisted of 2400 cGy cranial irradiation and concurrently six alternating injections of intrathecal methotrexate (12.5 mg/m^2^) and cytosine arabinoside (30 mg/m^2^). Maintenance therapy was given for 3 years and consisted of daily 6‐mercaptopurine, weekly methotrexate and monthly intrathecal therapy, with drug intensification comprising either vincristine, Adriamycin and I‐asparaginase (VAA) or cyclophosphamide, vincristine, cytosine arabinoside and prednisone (COAP).
Aken'ova	CML: Busulfan 4–6 mg orally daily or Endoxan tablets 50 mg daily. Splenic irradiation daily using Telecobalt 1.25 megavoltage beam
Omoti	NR
Chagaluka	ALL: Induction (4 weeks): Cyclophosphamide—IV 300 mg/m^2^ Day 1, Prednisolone—40 mg/m^2^/Day × 28 days PO, then wean for 5 days, Vincristine—IV 1.5 mg/m^2^ Day 1, 8, 15, 22, 29, Intrathecal methotrexate—Day 1, 7, 29, intrathecal. Setrin prophylaxis for pneumocytosis Maintenance (20 weeks): Vincristine—1.5 mg/m^2^ 4 weekly, Prednisolone—40 mg/m^2^ day × 5 days 4 weekly, 6‐mercaptopurine daily 60 mg/m^2^, Intrathecal methotrexate—4 weekly. Septrin prophylaxis for pneumocytosis
Chukwu	N/A
Sissolak^b^	CML: First‐line imatinib and second‐generation dasatinib and nilotinib
Faye^c^	CML: Imatinib
Tapela^d^	CML: Allopurinol, Imatinib
Rubagumya	ALL: Regimen 1 of Hunger Protocol [102] (developed for low‐resource settings), Vincristine, prednisone, cyclophosphamide, intrathecal methotrexate, 6‐mercaptopurine, dexamethasone and l‐asparaginase
Egesie	NR
Fentie^e^	CML: 400 mg Imatinib mesylate (Imatinib) as a daily dose
Aworanti	AML: Doxorubicin, cytosine arabinoside, COAP
Akaba	NR
Owojuyigbe	CML: Imatinib
Osei‐Owusu	ALL: l‐asparaginase
van Weelderen^f^	AML: Induction course 1 and 2: Doxurubicin (50 mg/m^2^, 4 h infusion, day 1,2,3), cytarabine (100 mg/m^2^, 1 h infusion, day 1–7), Triple intrathecal therapy (doses according to patients' age). Consolidation course 1 and 2: Etoposide (200 mg/m^2^, 1 h infusion, days 1–3), cytarabine (100 mg/m^2^, 1 h infusion, day 1–7), Triple intrathecal therapy—doses according to patients' age.
Shein	APL: Remission induction therapy—modified PETHEMA LPA99 protocol (117, 118) (with daunorubicin or doxorubicin replacing idarubicin); consolidation—three cycles of anthracycline chemotherapy and 2 years of maintenance ATRA and low dose chemo (methotrexate and 6‐mercaptopurine).
Olbara	NR
Kloopers	NR
Musaigwa	NR
Kasonkanji	ALL: Modified CALGB 10403 regimen for Malawi protocol [99].
Hodkinson	CLL: Imatinib, Dasatinib, Nilotinib
Dachi	NR
Jenkins	CN‐AML: Human Leukocyte Antigen‐matched allogenic stem cell transplant; non‐HLA matched were given High Dose Cytosine Arabinoside
Naicker	APL: ATRA, daunorubicin, anthracycline and corticosteroids (dexamethasone).
Vaughan	NR
Hodkinson	NR
Orido	NR

Abbreviations: APL, acute promyelocytic leukemia; h, hourly; NA, not applicable; NR, not reported.

^a^ CNS prophylaxis was only given to patients in whom complete hematological remission had been achieved.

^b^ Reported on the effect of BCR‐ABL 1 mutation on first‐line imatinib; some of the patients were moved to the second generation as there was full resistance based on the mutation.

^c^ 85.5% patients received hydroxyurea before imatinib treatment.

^d^ Twelve (27.9%) patients had been treated with imatinib before presentation at Rwinkwavu or Butaro hospitals. An additional 12 (27.9%) patients had received other treatment, primarily hydroxyurea, whereas the remaining 19 (44.2%) had never been treated for CML. All patients were initiated or continued on imatinib. During study follow‐up, reduced doses of imatinib ranged from 100 mg (*n* = 4, 9.5%), to 200 mg (*n* = 8, 19.1%), to 300 mg (*n* = 4, 9.5%). Four (9.5%) patients required dose increases to 600 mg. Indications for imatinib dose reduction: absolute neutrophil count (ANC) less than 1000/mL, or ·platelets less than 100 000/mL, or ·elevated liver function tests (LFTs), however, not more than twice the normal range. Indications for imatinib discontinuation: ANC less than 750/mL, or ·platelets less than 70 000/mL, or ·LFTs elevated beyond twice the normal range. Imatinib to be reintroduced when ANC rebounded to greater than 1000/mL, platelets greater than 75 000/mL^3^, and LFTs normalized.

^e^ 24.5% of patients were given a combination of hydroxyurea and imatinib.

^f^ The aim was to give chemotherapy every 4 weeks. During the study period, the infusion time of doxorubicin was increased from 1 to 4 h, the dose of etoposide was increased from 100 to 200 mg/m^2^, and the dose of cytarabine was reduced from 200 to 100 mg/m^2^ during consolidation. Triple intrathecal therapy included methotrexate, hydrocortisone, and cytarabine.

### 3.6 Survival Rate, Toxicity, Follow‐Up and Death

Survival data were available for 21 of the 32 included studies, with marked variability in follow‐up duration, survival rates, and reported mortality. Survival rates ranged from 2 weeks to 5 years (Table 6). Survival outcomes varied across leukemia subtypes. ALL showed survival durations ranging from 2 weeks to 2 years. AML had survival periods between 1 and 2 years, while APL ranged from 1 month to 3 years. Among chronic leukemias, CLL had a reported survival of approximately 7 months, whereas CML showed comparatively longer survival durations ranging from 18 months to 8 years. Survival outcomes ranged from short‐term responses (e.g., 50% survival at 2 weeks in Amsel [24]) to long‐term survival rates exceeding 5 years in more recent studies (e.g., 96‐month survival of 83.6% in Sissolak et al. [50]). Several studies reported encouraging survival durations among patients receiving modern treatment regimens. For instance, Shein et al. [49] reported a 3‐year survival rate of 76.5% in acute promyelocytic leukemia (APL) patients, while Faye et al. [30] observed an 81% survival rate at 2 years in chronic myeloid leukemia (CML) patients receiving imatinib. Similarly, Sissolak et al. [50] documented 92.9% and 83.6% survival at 60 and 96 months, respectively, among patients treated with tyrosine kinase inhibitors. Conversely, poorer survival outcomes were documented in settings with limited treatment access or intensive disease subtypes. Olbara et al. [43] reported a 3‐year survival rate of just 18%, and Rubagumya et al. [48] found a 71% mortality rate among children with acute lymphoblastic leukemia. Jenkins et al. [37] reported 1‐ and 2‐year overall survival rates of 36.4% and 24.2%, respectively, among patients with cytogenetically normal AML, despite intensive treatment including stem cell transplantation. Follow‐up duration varied significantly, with some studies offering only weeks to months of data (e.g., Chagaluka et al. [26]: 93–554 days), while others such as Kasonkanji et al. [38] followed participants for up to 7 years. In the nine [9] studies that reported treatment toxicity, the main variables reported were thrombocytopenia, neutropenia, and infections (Table 7). Loss to follow‐up was inconsistently reported; 59 patients were lost to follow‐up, and follow‐up time ranged from 11 months to 7 years. Leukemia‐related death rate from all studies was 10.76% (273 patients). (Table 6).

**TABLE 6:** Survival rates, follow‐up and death reported in selected studies.

First author	Survival rate	Loss of follow‐up	Follow‐up time	Number of deaths	Treatment‐related death	Death not related to treatment
Amsel	50% at 2 W	NR	NR	3	NR	NR
Kusman	30 M	4	11 M–20 M	17	17	NR
Jacobs	18 M–30 M	24	NR	58	NR	NR
Jacobs	NR	NR	NR	NR	NR	NR
Jacobs	C (59 W), N (39 W), T‐ALL (63 W), B‐ALL (13 W)	NR	260 W	2	2	NR
Aken'ova	48.7 M−53.3 M	NR	NR	25	15	10
Omoti	NR	NR	NR	NR	NR	NR
Chagaluka	93 D, 232 D, 554 D	NR	NR	17	NR	NR
Chukwu	NR	NR	NR	NR	NR	NR
Sissolak	60 M (92.9%); 96 M (83.6%)	NR	60.5 M		NR	NR
Faye	2 Y (81%)	6	3–210 M	9	NR	NR
Tapela	NR	4	58 M	NR	NR	NR
Rubagumya	8 persons (19%)	4	2Y	30 (71%)	NR	NR
Egesie	60.8 (SF)	NR	382D	NS	NR	NR
van Weelderen	2 Y (72.5%)	5	NR	NR	NR	NR
Shein	3 Y (76.5%)	8	35.4 M	18	5	13
Olbara	3 Y (18%)	NR	NR	41	27	14
Kloopers	NR	NR	NR	NR	NR	NR
Musaigwa	NR	NR	NR	NR	NR	NR
Fentie	NR	3	3 M	NR	NR	NR
Aworanti	NR	NR	NR	NR	NR	NR
Akaba	NR	NR	NR	NR	NR	NR
Owojuyigbe	NR	NR	NR	NR	NR	NR
Osei‐Owusu	NR	NR	NR	NR	NR	NR
Kasonkanji	50% at 12 M, 17% at 24 M	0	7Y	16	1	15
Hodkinson	7 M	1	1Y	16	NR	NR
Dachi	NR	NR	NR	NR	NR	NR
Jenkins	1‐ and 2‐year OS rates for all intensively treated patients were 36.4% (95% CI: 20.6–52.3) and 24.2% (95% CI: 11.4–39.6), respectively.	NR	239	12	12	0
Naicker	18 patients at 30 D	NR	NR	9	NR	NR
Vaughan	1.5 M to 5 M	NR	5 M	NR	NR	NR
Hodkinson	NR	NR	NR	NR	NR	NR
Orido	NR	NR	NR	NR	NR	NR

*Note:* D = days; M = months; NR = not reported; NS = not specified; SF = survival function; W = weeks; Y = years; treatment‐related death is defined as death caused as a result of treatment of leukemia (chemotherapy or radiotherapy).

**TABLE 7:** Treatment toxicity reported in selected studies.

First author	Treatment toxicity
Amsel	NR
Kusman	NR
Jacobs	NR
Jacobs	NR
Jacob	MR
Aken'ova	Septicemia, thrombocytopenia, infection
Omoti	NR
Chagaluka	NR
Chukwu	NR
Sissolak	NR
Faye	Grade 3 or 4 (anemia‐9.1; Neutropenia‐7.2; Thrombocytopenia‐1.8); Mouth ulcers‐7.2; Vomiting‐5.4; Drug eruption‐3.6; polyneuropathy‐1.8
Tapela	Neutropenia alone (5); Thrombocytopenia alone (5); Thrombocytopenia and neutropenia (2); Thrombocytopenia and anemia (1); Pancytopenia (2)
Rubagumya	NR
Egesie	NR
Fentie	Grade 3 or 4 thrombocytopenia (14). Neutropenia (7), bicytopenia (5), skin rash (3)
Aworanti	NR
Akaba	NR
Owojuyigbe	NR
Osei‐Owusu	High prothrombin time, activated partial thromboplastin time and D‐dimers. Low platelet count, protein C, protein S, and antithrombin III.
van Weelderen	NR
Shein	Differentiation syndrome‐1; sepsis‐1
Olbara	NR
Kloopers	NR
Musaigwa	NR
Kasonkanji	Induction: Grade 3 or 4 Anemia (1), Grade 3 or 4 neutropenia (9), Grade 3 or 4 thrombocytopenia (1), Constipation (*n* = 2), vision decreased (*n* = 1), epistaxis (*n* = 1), diarrhea (*n* = 1), urinary tract infection (*n* = 1), vomiting (*n* = 1). Remission consolidation: Grade 3 or 4 Anemia (2), Grade 3 or 4 neutropenia (3), Grade 3 or 4 thrombocytopenia (3), Diarrhea (*n* = 2), noncardiac chest pain (*n* = 1), lung infection (*n* = 1), epistaxis (*n* = 1), vomiting (*n* = 1). Interim Maintenance: Grade 3 or 4 Anemia (1). Delayed intensification: Grade 3 or 4 neutropenia (1), Grade 3 or 4 thrombocytopenia (1), Vision decreased (*n* = 1), nausea (*n* = 1), vomiting (*n* = 1). Maintenance: Grade 3 or 4 Anemia (1), Grade 3 or 4 neutropenia (3), Grade 3 or 4 thrombocytopenia (1), Diarrhea (*n* = 1), rectal hemorrhage (*n* = 1).
Hodkinson	Sepsis
Dachi	NR
Jenkins	52.4% of CN‐AML had resistant disease
Naicker	Differentiation syndrome
Vaughan	NR
Hodkinson	NR
Orido	NR

## 4 Discussion

### 4.1 Reported Cases of Leukemia and Subtypes

This systematic review synthesized findings from 32 studies, including 17 prospective and 15 retrospective designs, to establish the reported proportion of leukemia and subtypes, treatment regimens, survival rates, and follow‐up time. The overall reported cases of leukemia were found to be 2537, out of which 64.80% were acute and 35.20% were chronic. The proportion of reported cases of the subtypes were AML (947 cases, 37.33%), ALL (450 cases, 7.74%), CML (720 cases, 28.38%), CLL (173 cases, 6.82%), acute undifferentiated leukemia (2 cases, 0.08%), mixed phenotype acute leukemia (1 case, 0.04%), and unclassified leukemias (244 cases, 9.62%).

In a recent report from the American Cancer Society (ACS) [54] and National Cancer Institute (NCI) [55] in 2025, 66 890 new cases of leukemia were reported in the USA. In a 2025 report from ACS and NCI on leukemia in the USA, the estimated new cases were: AML: 22010 (32.9%), CLL: 23690 (35.8%), CML: 9560 (14.3%) cases, ALL: 6100 (9.1%) cases, and other leukemia: 5530 (8.3%) cases. In the same vein, the current report from the Cancer Research United Kingdom (CRUK) [56] and European Cancer Information System (ECIS) [57] in the United Kingdom showed that 10 000 people are diagnosed with leukemia annually in the UK. As at 2020 in the ECIS, the prevalent cases of the subtypes of leukemia were: AML (26 898 (36.9%) in the UK and Ireland, 33 969 (46.6%) in Southern Europe, 9813 (13.4%) in Spain and 2280 (3.1%) in Switzerland), CML (16 727 (38.0%) in the UK and Ireland, 18 852 (42.8%) in Southern Europe, 6953 (15.8%) in Spain and 1542 (3.5%) in Switzerland), CLL (73 383 (45.3%) in the UK and Ireland, 60 961 (37.6%) in Southern Europe, 21 235 (13.1%) in Spain and 6342 (3.9%) in Switzerland), and from CRUK statistics: ALL (765 cases each year (2017–2019) in the UK).

Comparing these statistics to findings from this systematic review, there is a low proportion of reported cases of leukemia and subtypes, which may not necessarily reflect the actual prevalence in sub‐Saharan Africa. The reported low cases may be attributed to challenges in diagnosis such as limited diagnostic tools, late‐stage diagnosis, and more importantly, no systems or statistical tools and software for reporting new cases [58]. The high number of leukemia cases reported from South Africa likely reflects its stronger healthcare infrastructure, better cancer surveillance systems, and greater research capacity compared with many African countries. Similarly, the relatively high case numbers from Nigeria and Kenya may be due to larger populations, increased awareness, and growing investment in leukemia research. In contrast, the lower number of reported cases from countries such as Ghana, Uganda, and Malawi may reflect underdiagnosis, limited diagnostic capacity, weak cancer registry systems, and lower research output rather than a truly lower leukemia burden. In a study conducted by Chukwu et al. [27], lag time was defined as the time between the onset of symptoms and diagnosis. The lag time for childhood cancers was 15.8 weeks, ALL was 4.2 weeks, AML was 14.3 weeks, and CML was 19.1 weeks. Although AML shares some clinical features with ALL, the longer delay may reflect differences in symptom progression or recognition by health professionals. Comparative analysis showed that diagnostic lag times for AML and CML were significantly longer than for ALL. These findings highlight the critical role of early symptom recognition and access to diagnostic services in improving timely diagnosis of leukemia in pediatric patients. Despite ALL's more favorable diagnostic timeline, delays in AML and CML remain concerning and suggest a need for targeted awareness among caregivers and frontline health providers. Only a few of the countries in the sub‐Saharan region do advanced testing to diagnose leukemia. However, even when advanced diagnostic techniques are available, these are quite expensive for the average person in the region. Apparently, most of the available blood count analyzers do not show blast cells, and standard stains are not available, making diagnosis more challenging. These factors, among others, contribute to the low prevalent cases reported in sub‐Saharan Africa [58]. Most patients die undiagnosed, and others are diagnosed at later stages of leukemia, which increases leukemia‐related death in sub‐Saharan Africa [59].

The observed survival patterns in this review demonstrate marked differences across leukemia subtypes, reflecting their distinct biological behavior and treatment responsiveness. Acute leukemias, particularly ALL and AML, showed relatively short survival durations, consistent with their aggressive clinical course and rapid disease progression when not promptly treated [60, 61]. Similarly, APL demonstrated variable survival outcomes, ranging from very short durations to longer survival, likely reflecting differences in early mortality and treatment response, particularly with the use of differentiation therapy such as all‐trans retinoic acid [61]. In contrast, chronic leukemias demonstrated comparatively longer survival. CML showed the longest survival range (up to several years), which is consistent with the transformative impact of tyrosine kinase inhibitor therapy, as highlighted in international clinical guidelines [62]. However, variability in survival likely reflects disparities in access to continuous therapy, monitoring, and healthcare infrastructure in resource‐limited settings. CLL showed relatively shorter survival in the included studies, which may be influenced by late diagnosis, limited access to modern targeted therapies, and variability in supportive care across study settings [63].

In Sub‐Saharan Africa, leukemia outcomes remain poor. As highlighted in this review, 273 of 2537 patients (10.76%) died, with diagnostic delays playing a significant role. Most patients are diagnosed at advanced stages, when curative treatment options are limited or unavailable. Factors such as limited access to diagnostics, late presentation, and unaffordable or inaccessible therapies—often stemming from systemic poverty—contribute to these outcomes. Consequently, five‐year survival rates are markedly low across the region. In contrast, developed countries benefit from early detection, specialized hematologic services, and access to advanced therapies, including targeted treatments and stem cell transplantation. As a result, 5‐year survival for childhood leukemia exceeds 85%, and outcomes for adults with acute leukemias have steadily improved due to molecularly tailored therapies and supportive care infrastructure [64, 65]. This stark disparity underscores the urgent need for investment in cancer care infrastructure, equitable access to diagnostics and therapeutics, and early referral systems across low‐resource settings.

### 4.2 Treatment of Leukemia

Treatment of leukemia includes chemotherapy, radiation therapy, immunotherapy, targeted therapy, stem cell transplantation, and supportive care. The common treatment options observed in this current review were chemotherapy, targeted therapy, and radiation therapy (commonly used where the leukemia has spread to the brain, spine, or other parts of the body). Chemotherapy, which is generally used, involves induction therapy, consolidation therapy, and maintenance therapy. In treating leukemia, induction therapy is initially administered to eligible patients to achieve complete remission (CR). Unfortunately, recurrence will unavoidably occur if treatment is stopped because minimal residual disease frequently persists in complete remission (CR). Therefore, consolidation therapy should be used after a positive response to induction therapy to eliminate any remaining disease and ensure long‐lasting remission. Currently, chemotherapy and radiation therapy are used in conjunction when leukemic cells infiltrate the central nervous system, spleen, and other organs [66].

#### 4.2.1 Treatment of AML

Studies [24, 25, 37, 52] that reported on AML in the present review used different therapy protocols. The mainstay of induction therapy consists of the “7 + 3” regimen, which combines 7 days of continuous infusion cytarabine with 3 days of anthracycline. Standard dosing of cytarabine consists of 100–200 mg/m^2^ daily administered as a continuous infusion over 7 days. A bone marrow aspirate and core biopsy should be performed 14 days following the start of treatment to assess the response to induction therapy [67]. With induction therapy, around 60%–80% of individuals with de novo AML will eventually achieve CR [68]. Consolidation therapy should be made available to patients who are in remission in order to eliminate any remaining disease and stop relapses. Chemotherapy and allogeneic hematopoietic stem cell transplantation (allo‐HSCT) are viable treatments for consolidation.

Chimeric antigen receptor‐T (CART) therapy, Monoclonal antibodies, FLT3‐ITD inhibitors, and STAT inhibitors are alternative, cutting‐edge AML therapeutic alternatives. Synthetic T‐cell receptors that resemble antibodies are known as chimeric antigen receptors. They fuse the intracellular and transmembrane domains of a T‐cell receptor with a single‐chain variable fragment from a monoclonal antibody. This makes it possible to produce a population of chimeric antigen receptor‐T (CART) cells from the host that may be targeted against a specific antigen. Recently, Kenderian et al. [69] developed a CD33‐specific CAR using gemtuzumab ozogamicin's single‐chain variable fragment. Such CD33‐specific CART cells have been shown to exhibit strong in vitro activity against AML cell lines [70]. For patients with relapsed or refractory AML, CART cell therapy may offer an alternate treatment option, albeit it is still in its early stages of clinical trials [71].

Monoclonal antibodies exert their anti‐tumor activity through direct antibody‐dependent cytotoxicity or through the conjugation of cytotoxic agents, which allows for the targeted delivery of potent chemotherapy to neoplastic cells. A humanized recombinant antibody called gemtuzumab ozogamicin (GO) targets the transmembrane protein CD33, which is expressed on myeloid‐lineage cells [72]. The antibody is internalized by CD33‐positive cells after conjugating with the DNA‐cleaving lethal drug calicheamicin [73, 74]. The powerful microtubule‐disrupting drug monomethyl auristatin E (MMAE) is coupled with the completely human anti‐CD37 IgG antibody AGS67C to form AGS67E [75]. AGS67E triggers apoptosis and enables the targeted distribution of MMAE to malignant cells that express CD37. Even though CD37 is not expressed on normal myeloid stem cells, it was recently shown that CD37 is differentially expressed on the surface of CD34+/CD38‐AML stem cells. Seven out of 16 AML cell lines showed cytotoxicity, changed cell development, and death after leukemic cells were treated in vitro with nanomolar concentrations of AGS67E. In a mouse xenograft model of AML, it was also discovered that administering AGS67E significantly reduced tumor engraftment, leading to undetectable leukemic cell levels in three of four AML samples [75]. Therefore, CD37 might be a new therapeutic target for the targeted suppression of leukemic cell proliferation. Even though AGS67E is still in the early stages of clinical testing, it is a potential new treatment.

Because of the high frequency of FLT3 mutations and their influence on prognosis, suppression of this tyrosine kinase (TK) has long been identified as a possible therapeutic target in AML. Tested agents of FLT3‐ITD inhibitors include the first‐generation inhibitors sorafenib [76, 77, 78, 79] and midostaurin [80, 81, 82, 83, 84], as well as newer second‐generation agents such as quizartinib [85, 86, 87] and crenolanib [88, 89].

Up to 50% of AML cases had increased STAT3 tyrosine phosphorylation, which is associated with a poorer prognosis. The FLT3 receptor ligand also stimulates the STAT3 signaling pathway [90], which may be a crucial stage in the emergence of FLT3 TKI resistance. A number of small STAT3 inhibitor compounds have been created and are presently being researched for the treatment of AML. Redell et al. (2011) demonstrated that AML cell lines treated with the STAT3 inhibitor C188‐9 [91] had reduced STAT3 phosphorylation and increased apoptosis. In AML cell lines grown with bone marrow stromal cells present, the optimized chemical MM‐206 more recently showed a dose‐dependent activation of apoptosis. By decreasing the number of blasts and increasing survival in AML‐engrafted mice, MM‐206's anti‐tumor action was validated in vivo [92].

For patients with acute myeloid leukemia (AML) who are ineligible for intensive chemotherapy due to age or comorbidities, venetoclax combined with azacitidine has become a key treatment option [93]. The VIALE‐A trial demonstrated that this regimen significantly improves outcomes compared to azacitidine alone. In the final analysis, median overall survival was 14.7 months with venetoclax‐azacitidine versus 9.6 months with placebo‐azacitidine, with a hazard ratio of 0.58 (*p* < 0.001). The combination also resulted in higher complete remission or remission with incomplete hematologic recovery (CR/CRi) rates (66.8% vs. 29.0%) [93]. This approach provides a viable standard‐of‐care alternative for frail or elderly AML patients.

#### 4.2.2 Treatment of APL

In the present study, the main treatment of APL was all‐trans retinoic acid (ATRA) as reported in two studies [42, 49]. Acute promyelocytic leukemia (APL) is a highly curable subtype of acute myeloid leukemia. Treatment has evolved significantly from chemotherapy alone to targeted therapies. The standard first‐line treatment now combines ATRA with arsenic trioxide (ATO), especially for low‐ and intermediate‐risk patients (WBC < 10 000/μL), eliminating the need for traditional cytotoxic chemotherapy in many cases [94]. This combination has shown superior outcomes compared to ATRA plus chemotherapy, with >90% complete remission rates and overall survival exceeding 90%. For high‐risk patients (WBC > 10 000/μL), ATRA/ATO is insufficient alone. Regimens may incorporate idarubicin or gemtuzumab ozogamicin (GO) to achieve cytoreduction and mitigate risks like differentiation syndrome and coagulopathy [94, 95]. Supportive care—including early ATRA administration, platelet and fibrinogen transfusions, and prompt management of differentiation syndrome with corticosteroids—is critical to reduce early mortality. Maintenance therapy remains debated. Some trials support ATRA‐based maintenance, especially in high‐risk cases, while others find no added benefit if molecular remission is achieved [95]. Overall, APL represents a paradigm shift in leukemia therapy with curative outcomes driven by molecularly targeted approaches.

#### 4.2.3 Treatment of CML

The common therapy found in this review was imatinib [23, 30, 31, 35, 47, 50, 51]. A combination of imatinib and second‐generation dasatinib and nilotinib was used in one study [50], and busulphan therapy was utilized in two studies [23, 35]. In a systematic review conducted by Ferdinand et al. [96], in the United Kingdom, the chemotherapy reported as first‐line therapy were Imatinib [Chronic Phase (CP) standard dose 400 mg Once Daily (OD) up to 400 mg twice daily (BID); Accelerated Phase (AP) 600 mg OD up to 400 mg BID], Dasatinib (CP 100 mg/day; CP 70 mg BID), Nilotinib (400 mg BID) and Bosutinib (standard dose, 500 mg/day), second‐line therapy [Imatinib, imatinib‐intolerant: dasatinib and nilotinib, Imatinib resistance: dasatinib and nilotinib, Imatinib resistance: bosutinib], and third‐line therapy [Dasatinib, Nilotinib, Bosutinib]. Comparing these findings to treatment protocols in sub‐Saharan Africa, Imatinib therapy is the standard therapy, but in cases of imatinib intolerance or resistance, other therapies such as dasatinib, bosutinib, or nilotinib in combination with imatinib can be used. Imatinib doses of 600 mg or even 800 mg per day are typically used to treat individuals who present in advanced stages of CML; nevertheless, the outcomes are significantly worse than those of patients in CP who receive a lower dose. Advanced phase disease is incredibly diverse in practice. Patients with accelerated phase CML may have a disease that is just marginally more advanced than late‐chronic‐phase CML, and they may benefit from long‐term effects from imatinib alone [97]. The management of CML has been significantly improved by the introduction of tyrosine kinase inhibitors (TKIs), particularly the availability of generic formulations. The first‐generation TKI, Imatinib, is widely available as a generic (imatinib mesylate), which has greatly reduced treatment costs and expanded access globally following patent expiry. In addition, second‐generation TKIs such as Dasatinib and Nilotinib are increasingly available in generic forms in several low‐ and middle‐income countries, further improving therapeutic options for CML patients. These advances have been shown to enhance affordability and access to long‐term therapy, particularly in resource‐limited settings [62, 98]. However, despite the growing availability of generics, access in sub‐Saharan Africa remains inconsistent due to differences in procurement systems, regulatory frameworks, and healthcare financing, which continue to affect equitable distribution and sustained treatment adherence.

#### 4.2.4 Treatment of ALL

Therapy Protocols found in this review were the Modified CALGB 10403 regimen for Malawi protocol [99], St Jude regimen [100], modified LSA‐L2 protocol [101], Hunger Protocol [102], and other unnamed ones. The therapies found in these protocols were similar. Therapy regimens such as vincristine, prednisone, cyclophosphamide (Cy), Doxorubicin, and l‐asparaginase were used in the induction, consolidation, and maintenance stages of these protocols. In a randomized trial study conducted by Annino et al. [103], the *GIMEMA ALL 0288* treatment plan was utilized. Pretreatment with prednisone (PDN) was administered in increasing doses over a period of 7 days as 20 mg/m^2^ on day −7, 30 mg/m^2^ on day −6, 40 mg/m^2^ on day −5, and 60 mg/m^2^ on days −4 to −1. Both a 5‐drug regimen consisting of Cy, vincristine (VCR), daunorubicin (DNR), asparaginase (ASP), and prednisone (PDN) or a 4‐drug regimen consisting of VCR, DNR, ASP, and PDN were administered during the first induction phase over a period of 4 weeks. Day 32 saw the evaluation of CR. Patients who were not in CR were given a salvage regimen that includes mitoxantrone (MITOX) and high‐dose cytosine‐arabinoside (HDARA‐C) in continuous infusion. After receiving induction phase II (VCR, MITOX, and PDN) or salvage chemotherapy, patients in CR who had completed induction phase I received intensification, which included three cycles of L‐VAMP and four doses of teniposide (VM‐26) and ARA‐C. Combining VCR, methotrexate (MTX), ARA‐C, and dexamethasone (Dexa) results in L‐VAMP. After that, folinic acid was given as a rescue. L‐VAMP was offered every 15 days. The two options for post‐intensification therapy were maintenance alone or consolidation plus maintenance. Eight medications were used in consolidation therapy, which was divided into five courses [A(Cy + ARA‐C), B(VCR + DNR + PDN), C(VCR + MITOX+PDN), D(VM‐26 + ARA‐C), and E(L‐VAMP)] and repeated three times each. Six months was the anticipated time for consolidation. For 3 weeks, the maintenance chemotherapy consisted of weekly intramuscular MTX and daily oral 6‐mercaptopurine (6‐MP), followed by VCR and PDN pulses. The entire post‐intensification treatment period lasted 24 months. In addition to the systemic high‐dose MTX administered during the three courses of L‐VAMP, the central nervous system (CNS) prophylactic strategy involved weekly intrathecal MTX + PDN r during induction (days 0, 8, 15, 22), monthly during intensification, and post‐intensification for a total of 16 doses. Also, CD19‐directed chimeric antigen receptor‐T (CART) cell therapy has demonstrated promising results in the management of acute lymphoblastic leukemia [104, 105, 106].

#### 4.2.5 Treatment of CLL

There is no need for treatment or risk assessment when CLL is identified at an early stage of the disease, as defined by Rai or Binet [107] (Binet A and B or Rai 0, I, and II without active disease) [108, 109]. Patients should be monitored every 3 months for the first year, and the disease stage should then be dynamically adjusted [110]. Because early chemotherapy treatment with fludarabine or chlorambucil does not lead to a longer overall survival, this “watch and wait” strategy is warranted and recommended [111, 112].

First‐line therapy involves a regimen that includes either a B‐cell leukemia/lymphoma 2 (BCL2) inhibitor (venetoclax) or a covalent Bruton tyrosine kinase (BTK) inhibitor (acalabrutinib, zanubrutinib, or ibrutinib) [113]. Venetoclax is administered as a first‐line treatment for 1 year in conjunction with obinutuzumab, a monoclonal anti‐CD20 antibody (overall survival, 82% at 5‐year follow‐up). Following the failure of venetoclax and covalent BTK inhibitors, pitobrutinib, a noncovalent BTK inhibitor, has demonstrated an overall response rate of over 70%. Idelalisib and duvelisib, two phosphoinositide 3′‐kinase (PI3K) inhibitors, can be recommended for diseases that worsen when BTK inhibitors and venetoclax are used; however, patients need to be closely watched for side effects such as infections and autoimmune diseases. Recently, chimeric antigen receptor‐engineered (CAR)‐T cell immunotherapy emerged as a new treatment option for CD19+ B cell malignancies [114]. Lisocabtagene maraleucel, used in chimeric antigen receptor T‐cell (CAR‐T) therapy, was linked to a 45% complete response rate in individuals who experienced several relapses. Allogeneic hematopoietic cell transplantation is the sole treatment for CLL that may be effective, and it is still a possibility following the use of targeted medicines [115].

## 5 Limitation, Recommendations, and Conclusion

### 5.1 Limitation

Some countries or regions within Sub‐Saharan Africa may be underrepresented, leading to a potential bias in the data. Prevalence statistics may be lacking or untrustworthy in a number of countries due to the lack of strong population‐based studies and cancer registries. In addition, the studies included in the review may have varied in terms of methodology, population characteristics, diagnostic methods, and treatment protocols, which could impact the generalizability of the findings. Lastly, readers should interpret these findings with consideration of the potential for publication bias, selection bias arising from the study eligibility criteria, and reporting bias within the included studies.

### 5.2 Recommendations

Enhancing laboratory and diagnostic facilities should be a priority for governments and international organizations, particularly in underserved and rural areas, including providing access to contemporary diagnostic tools such as molecular diagnostics, cytogenetic testing, and flow cytometers. Continuous professional development programs should be implemented to train healthcare providers in early recognition and accurate diagnosis of leukemia. Efforts should be made to reduce the costs of chemotherapy drugs, supportive medications, and other cancer therapies. Governments and NGOs could collaborate with pharmaceutical companies to negotiate reduced prices for essential cancer medications or offer subsidies for low‐income patients. A more coordinated approach to healthcare system strengthening is needed, including investment in health infrastructure, human resources, and policy development to ensure better care for cancer patients. Furthermore, governments should prioritize cancer as part of their national health agendas, particularly in countries with rising cancer burdens. Public health campaigns aimed at educating the population about leukemia prevention, early signs, and treatment options could help in reducing diagnostic delays and improving survival rates. More robust, population‐based studies and cancer registries are needed to provide accurate data on leukemia prevalence, outcomes, and treatment efficacy in Sub‐Saharan Africa. This would help inform national health strategies and policy decisions. Local researchers should be encouraged to conduct studies on leukemia treatment protocols that are culturally and economically feasible in sub‐Saharan Africa. This includes research into cheaper, more accessible treatment options and exploring local biological factors that might influence leukemia in the region.

### 5.3 Conclusion

Of the 2537 leukemia cases that were recorded overall, 64.80% were acute, and 35.20% were chronic. This systematic review highlights the significant burden of leukemia in the region, with acute forms being more prevalent than chronic leukemias. Acute myeloid leukemia (AML) emerged as the most reported subtype, followed by chronic myeloid leukemia (CML). Although survival extended up to 5 years in some patients, the overall outcomes remained poor, with notable mortality and loss to follow‐up. The variability in survival and follow‐up durations underscores the need for improved diagnostic capacity, standardized treatment protocols, and robust patient monitoring systems. Strengthening cancer registries and enhancing access to specialized care are essential to improving survival outcomes for leukemia patients.

## Author Contributions

**Linda K. Olaga:** writing – review and editing. **Stephen Twumasi:** conceptualization, investigation, visualization, validation, methodology, writing – review and editing, software, project administration, resources, supervision, data curation, writing – original draft, formal analysis. **Samuel Essien‐Baidoo:** writing – review and editing. **Daniel N. M. Antonio:** writing – review and editing. **Richard O. Ansah:** writing – review and editing. **Allwell A. Ayirebi:** conceptualization, methodology, writing – original draft, validation, visualization, writing – review and editing, project administration, supervision, data curation, investigation, software, resources. **Benedict Sackey:** writing – review and editing. **John A. Sah:** writing – review and editing. **Eugene A. Ansah:** writing – review and editing. **Yvonne Anang‐Quartey:** writing – review and editing. **Enoch O. Anto:** writing – review and editing. **Wina I. O. Boadu:** writing – review and editing. **Antoinette Koomson:** writing – review and editing. **Ama G. Boadu:** writing – original draft, writing – review and editing. **Lilian A. Boateng:** writing – review and editing, writing – original draft. **Godfred A. Donkor:** writing – review and editing. **Angela Opoku:** writing – review and editing. **Paulina Duah:** writing – review and editing.

## Funding

The authors have nothing to report.

## Disclosure

A preprint has previously been published [116].

## Ethics Statement

The authors have nothing to report.

## Consent

The authors have nothing to report.

## Conflicts of Interest

The authors declare no conflicts of interest.

## Data Availability

The data that supports the findings of this study are available in the supplementary material of this article.

## Search Strategy for Studies on Acute and Chronic Leukemia in Sub‐Saharan Africa (Searches Were Conducted From Database Inception to 15 January 2025)

### PubMed/Medline

(“Leukemia”[Mesh] OR leukemia[tiab] OR leukemias[tiab] OR “acute leukemia”[tiab] OR “acute myeloid leukemia”[tiab] OR AML[tiab] OR “acute lymphoblastic leukemia”[tiab] OR ALL[tiab] OR “chronic leukemia”[tiab] OR “chronic myeloid leukemia”[tiab] OR CML[tiab] OR “chronic lymphocytic leukemia”[tiab] OR CLL[tiab]) AND (“Africa South of the Sahara”[Mesh] OR “sub‐Saharan Africa”[tiab] OR “West Africa”[tiab] OR “East Africa”[tiab] OR “Southern Africa”[tiab] OR “Central Africa”[tiab] OR Africa[tiab]) AND (treatment[tiab] OR therapy[tiab] OR chemotherapy[tiab] OR radiotherapy[tiab] OR “stem cell transplantation”[tiab] OR “hematopoietic stem cell transplantation”[tiab] OR “bone marrow transplantation”[tiab] OR remission[tiab] OR survival[tiab] OR mortality[tiab] OR morbidity[tiab] OR “quality of life”[tiab]).

### Scopus

TITLE‐ABS‐KEY (leukemia OR “acute leukemia” OR “acute myeloid leukemia” OR AML OR “acute lymphoblastic leukemia” OR ALL OR “chronic leukemia” OR “chronic myeloid leukemia” OR CML OR “chronic lymphocytic leukemia” OR CLL) AND TITLE‐ABS‐KEY (Africa OR “sub‐Saharan Africa” OR “West Africa” OR “East Africa” OR “Southern Africa” OR “Central Africa”) AND TITLE‐ABS‐KEY (treatment OR therapy OR chemotherapy OR radiotherapy OR “stem cell transplantation” OR “bone marrow transplantation” OR remission OR survival OR mortality OR morbidity OR “quality of life”).

### Google Scholar

Searches were conducted using combinations of the following search phrases:

“acute leukemia” “sub‐Saharan Africa”.

“chronic leukemia” “sub‐Saharan Africa”.

“acute myeloid leukemia” Africa treatment.

“acute lymphoblastic leukemia” Africa survival.

“chronic myeloid leukemia” Africa chemotherapy.

“chronic lymphocytic leukemia” Africa outcomes.

### Web of Science

TS = (leukemia OR “acute leukemia” OR “acute myeloid leukemia” OR AML OR “acute lymphoblastic leukemia” OR ALL OR “chronic leukemia” OR “chronic myeloid leukemia” OR CML OR “chronic lymphocytic leukemia” OR CLL) AND TS = (Africa OR “sub‐Saharan Africa” OR “West Africa” OR “East Africa” OR “Southern Africa” OR “Central Africa”) AND TS = (treatment OR chemotherapy OR radiotherapy OR “stem cell transplantation” OR remission OR survival OR mortality OR morbidity OR “quality of life”).

### Embase

(‘exp leukemia’/exp. OR leukemia:ti,ab OR ‘acute leukemia’:ti,ab OR ‘acute myeloid leukemia’:ti,ab OR AML:ti,ab OR ‘acute lymphoblastic leukemia’:ti,ab OR ALL:ti,ab OR ‘chronic leukemia’:ti,ab OR ‘chronic myeloid leukemia’:ti,ab OR CML:ti,ab OR ‘chronic lymphocytic leukemia’:ti,ab OR CLL:ti,ab) AND (‘africa south of the sahara’/exp. OR ‘sub‐Saharan Africa’:ti,ab OR Africa:ti,ab) AND (treatment:ti,ab OR chemotherapy:ti,ab OR radiotherapy:ti,ab OR ‘stem cell transplantation’:ti,ab OR survival:ti,ab OR mortality:ti,ab OR morbidity:ti,ab).

### Cochrane Library

(Leukemia OR “acute leukemia” OR “chronic leukemia” OR AML OR ALL OR CML OR CLL) AND (Africa OR “sub‐Saharan Africa”) AND (treatment OR chemotherapy OR radiotherapy OR transplantation OR survival).

### African Journals Online (AJOL)

Searches were performed using the AJOL search interface with the following combinations:

leukemia AND Africa.

acute leukemia AND Africa.

chronic leukemia AND Africa.

acute myeloid leukemia.

acute lymphoblastic leukemia.

chronic myeloid leukemia.

chronic lymphocytic leukemia.

leukemia chemotherapy Africa.

leukemia survival in Africa.
